# Health beliefs model to explore older adults’ dementia prevention and health promotion from 2021 to 2022 in Taiwan: A cross-sectional survey study

**DOI:** 10.1097/MD.0000000000039744

**Published:** 2024-10-18

**Authors:** Fu-Ju Tsai, Sheng-Wei Shen, Yih-Jin Hu, Chie-Chien Tseng

**Affiliations:** a Department of Nursing, Fooyin University, Taiwan ROC; b National Taiwan Normal University, Taiwan ROC; c Emory University, A; d Department of Neurology, Pingtung Hospital, Ministry of Health and Welfare, Taiwan, ROC; e Chung Shan Medical University, Taiwan ROC; f Department of Health Promotion and Health Education, National Taiwan Normal University, Taiwan ROC; g National Taiwan Normal University, Taiwan ROC; h Department of Health Promotion and Health Education, National Taiwan Normal University, Taiwan ROC; i University of Florida, A.

**Keywords:** dementia prevention, health beliefs model, health promotion, older adults

## Abstract

One person suffers from dementia every 3 seconds globally. Thirteen older adults aged 65 and older will have dementia, and 1 in 5 older adults over the age of 80 years will have dementia in Taiwan. Older adults should be equipped with demonstrated health beliefs regarding dementia prevention and health promotion about Ascertain Dementia 8-item Questionnaire (AD8), cues to action, health beliefs, self-efficacy, and behavioral intention in daily life. The purpose of this study was to survey older adults’ demographic background, AD8, cues to action, health beliefs, self-efficacy, and behavioral intention for dementia prevention and health promotion. A cross-sectional survey design was used. Convenience sampling was performed. A total of 330 older adults participated in the study. The questionnaire used in this study included questions on older adults’ demographic background, AD8, cues to action, health beliefs, self-efficacy, and behavioral intention. The researcher collected complete data by receiving the sampling on paper or by interview from October 8, 2021, to February 12, 2022. The SPSS 23.0 statistical package was employed for quantitative analysis. Data analysis included frequency, percentage, mean, standard deviation (SD), Spearman’s rho correlation, and simple regression analysis. The findings showed that older adults had the following mean scores on health beliefs (perceived susceptibility 13.45 ± SD 2.34, perceived severity 13.54 ± SD 2.69, perceived benefits 16.57 ± SD 2.84, perceived barriers 8.20 ± SD 3.69), self-efficacy 16.96 ± SD3.52, and behavioral intention 19.56 ± SD 3.51. Older adults’ demographic background, perceived susceptibility, perceived severity, perceived benefits, perceived barriers, and self-efficacy explained 56.1% of the variance in behavioral intention. The conclusions of the study indicated that older adults’ demographic background, AD8, cues to action, health beliefs, self-efficacy, and behavioral intention constituted the main factors for effective dementia prevention and health promotion. In the future, the research team will continue to explore older adults’ dementia prevention and develop many strategies on health promotion, as well as slowing the aging brain process.

## 1. Introduction

Globally, 1 person suffers from dementia every 3 seconds in the world. Thirteen older adults aged 65 and older will have dementia, and 1 in 5 older adults over the age of 80 years will have dementia in Taiwan.^[1]^ In the importance of dementia prevention and health promotion, medical-care members will concern on this work to help older adults with developing dementia patients and families in the treatment process. Dementia may impact on the patient’s individual life and family lives. As far as society, people should have the concepts of dementia prevention and health promotion to care about dementia patients among older adults. In health-care systems, a team group should be nice and patience to treat older adults with dementia patients. People get older and older with developing dementia, and it effects individual, family, society, and health-care systems. Therefore, older adults will need to have health beliefs, prevent dementia, and promote health in the future life.

Health beliefs consist of perceived susceptibility, perceived severity, perceived benefits, and perceived barriers to play a role with health promotion for preventing diseases. Dementia prevention may use the health beliefs model to prevent older adults getting older for developing dementia. Another, Ascertain Dementia 8-item Questionnaire (AD8) is a tool to screen early dementia development. Thus, older adults should be equipped with a demonstrated health belief model for dementia prevention and health promotion in terms of AD8,^[2]^ cues to action, health beliefs, self-efficacy, and behavioral intention in daily life. In dementia prevention and health promotion, a health belief model constitutes an important development on perceived susceptibility, perceived severity, perceived benefits, and perceived barriers for health beliefs and self-efficacy with healthy lifestyles, especially behavioral intentions to promote quality of life.^[3]^

In terms of dementia prevention and health promotion, 5 factors are considered: facial smile, thanks, eye contact, needing the moment, and patience for listening.^[4]^ Dementia prevention aims to improve older adults’ quality of life and promote behavioral changes in the physical, mental, spiritual, and social health promotion.^[5,6]^ Dementia prevention is established on knowledge, positive beliefs, health attitudes, and improved quality of life for health promotion.^[7]^ In addition, laughter may increase dementia prevention and health promotion on social interactions for healthy behaviors in daily life.^[8]^ Older adults’ dementia prevention and health promotion comprise long-term engagement in numerous factors, including cognitive activities, social stimulation, lifestyle changes, a balanced diet, smoking cessation, physical activities, and listening to music.^[9]^ Dementia prevention also requires modification of unhealthy lifestyles related to obesity, smoking, excessive alcohol,^[10]^ physical inactivity,^[11]^ emotional problems with depression,^[12]^ and the 3 major problems of hypertension, diabetes mellitus, and dyslipidemia.^[13,14]^ Therefore, dementia prevention aims to increase motivation, change lifestyles, and build behavioral intentions for health promotion.^[15]^

Older adults may develop meaningful cognitive functions to delay the aging brain and promote their health.^[16]^ Behavioral change resulting from cognitive stimulation interventions improves cognitive, physical, and social activities to achieve and maintain healthy aging among older adults.^[17]^ For example, using a smartphone can provide older adults with the ability to master simple calculation and memory tasks, and cognitive tasks can be performed while walking.^[17]^ Cognitive stimulation has been shown to increase dementia prevention and promote several healthy lifestyle habits.^[18]^ Health education, social interaction, physical activity, cognitive activity, and emotional well-being are the primary modalities for promoting older adults’ chronic health conditions associated with dementia prevention and health promotion.^[19]^

Cholesterol levels in older adults in high-density lipoprotein and low-density lipoprotein cholesterol are associated with diminished cognitive function; thus, it is essential to modify increased high-density lipoprotein and decreased low-density lipoprotein for health promotion and dementia prevention.^[20]^ Fruits and vegetables have been shown to contribute significantly to dementia prevention in terms of cognitive function and mental health promotion.^[21]^ The “Mediterranean Diet” has also been demonstrated to dementia prevention and support health promotion.^[22]^ Physical activity is another highly effective way to obtain cognitive benefits for dementia prevention.^[23,24]^ The regular activity of leaving one’s home to shop for food is also confirmed to be correlated with health promotion and dementia prevention.^[25]^ Furthermore, social and emotional support are reported to be associated with dementia prevention and health promotion in older adults.^[26]^

Therefore, older adults should be provided with AD8 screening, cues to action, health beliefs (perceived susceptibility, perceived severity, perceived benefits, and perceived barriers), self-efficacy, and behavioral intention regarding dementia prevention and health promotion globally as well as in many communities in Taiwan.

## 2. Purpose

The purpose of this study was to survey older adults’ demographic background, AD8, cues to action, and health belief model on perceived susceptibility, perceived severity, perceived benefits, perceived barriers, self-efficacy, and behavioral intention for dementia prevention and health promotion from 2021 to 2022.

## 3. Methods

### 3.1. Design

A cross-sectional survey design was used in the study.

### 3.2. Framework

**Figure 1.**
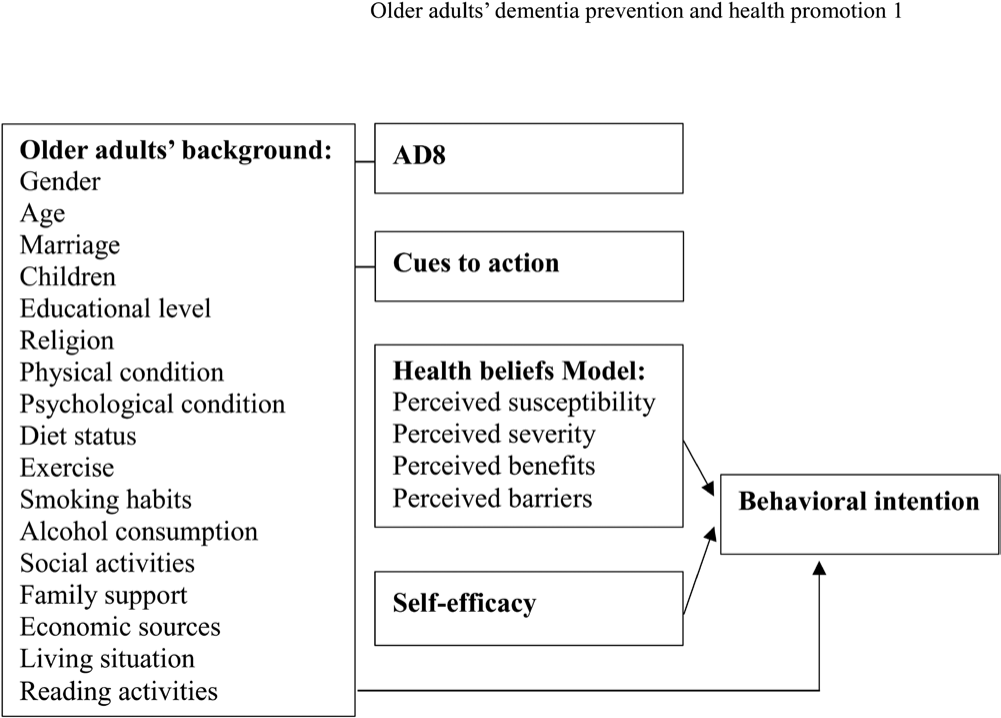
The framework of this study included older adults’ background, AD8, cues to action, health beliefs model, and self-efficacy relationships between behavioral intention.

The framework of this study comprised older adults’ background, AD8, cues to action, health beliefs model, and self-efficacy relationships with behavioral intention (Fig. 1). The background of the older adults included gender, age, marriage, children, educational level, religion, physical condition, psychological condition, diet status, exercise, smoking habits, alcohol consumption, social activities, family support, economic sources, living situation, and reading activities (Fig. 1). Furthermore, the framework also comprised AD8, cues to action, a health belief model (perceived susceptibility, perceived severity, perceived benefits, and perceived barriers), self-efficacy, and behavioral intention (Fig. 1). This survey study on dementia prevention and health promotion explored older adults’ background, AD8, cues to action, health beliefs model, and self-efficacy related to behavioral intention (Fig. 1).

### 3.3. Participants

The survey study used G Power software with the following parameters: effect size = 0.25, α = 0.05, power = 0.8, and final total sample size = 330. The inclusion criteria of this study were recruited 55-year-old or older community residents, and the exclusion criteria was not recruited dementia patients in Kaohsiung-Pingtung City, Taiwan, ROC. Convenience sampling was used in the study to receive the sampling on paper or by interview. A small number of older adults had to assist in the interview in the sampling process. A total of 330 older adults participated in this survey on dementia prevention and health promotion.

### 3.4. Ethical considerations

This cross-sectional survey study on dementia prevention and health promotion in older adults was approved by the Institutional Review Board/Ethics Committee of National Taiwan Normal University (IRB No. 202108EM023) on September 5, 2021.

### 3.5. Instruments

AD8 was confirmed for use by Yang.^[2]^ AD8 constitutes an effective screening tool for dementia prevention and community intervention to promote health in older adults.^[27]^ The AD8 is a sensitive approach to identify cognitive decline worldwide^[28]^ and has been demonstrated to be effective in screening older adults for dementia prevention.^[29]^ The AD8 is a brief self-report tool developed in Taiwan^[30]^ that may identify early cognitive impairment for dementia prevention.^[31,32]^ AD8 cognitive screening is useful for dementia detection in older adults.^[33,34]^ AD8 positive screen has the strongest association among older adults aged 65–75 for early-stage dementia diagnosis,^[35]^ and it may also identify early mild dementia.^[36]^

The tools of this study used a self-designed questionnaire with AD8 and a health beliefs model for dementia prevention and health promotion. The questionnaire of the study included older adults’ demographic background, AD8, cues to action, health beliefs model (perceived susceptibility, perceived severity, perceived benefits, and perceived barriers), self-efficacy, and behavioral intention. The content validity index of the questionnaire with 3 scholars and experts was 0.96. The Cronbach’s α reliabilities of the preliminary test with 33 older adults in communities were as follows: AD8 0.90 (1–8 items); perceived susceptibility 0.74 (9–12 items); perceived severity 0.78 (13–16 items); perceived benefits 0.73 (17–20 items); perceived barriers 0.91 (21–24 items); self-efficacy 0.90 (25–29 items); and behavioral intention 0.94 (30–34 items).

### 3.6. Data collection

The researcher explained the questionnaire to the participants and provided them with a small gift to encourage their participation. A total of 400 older adults participated voluntarily in the study and answered the “Health Beliefs Model on Dementia Prevention and Health Promotion” questionnaire. Finally, 330 (82.5%) questionnaires were completed by the older adults. Seventy (17.5%) questionnaires contained missing or nonresponse data. Older adults did not care about dementia prevention and health promotion in Kaohsiung-Pingtung City, Taiwan, ROC. It was hard to collect the survey questionnaire. In aggregate, the researcher collected complete data by receiving the sampling on paper or by interview from October 8, 2021, to February 12, 2022.

### 3.7. Data analysis

The SPSS 23.0 statistical package was employed for quantitative analysis. Data analysis included frequency, percentage, mean, SD, Spearman’s rho correlation, and simple regression analysis.

## 4. Results

### 4.1. Older adults’ background

The older adults’ background of the study is indicated in Table 1.

**Table 1:** Older adults’ background.

N = 330	Items	Frequency	Percentage
Gender	1. Male 2. Female	140 190	42.4 57.6
Age	1. 55–65 years old 2. 66–5 years old 3. Older than76 years	85 136 109	25.8 41.2 33.0
Marriage	1. Unmarried 2. Married 3. Widowed	9 222 99	2.7 67.3 30.0
Children	1. No children 2. Children	4 326	1.2 98.8
Educational level	1. 6 > years 2. 6–12 years 3. 12 < years	39 245 46	11.8 74.2 13.9
Religion	1. No religious beliefs 2. Religious beliefs	56 274	17.0 83.0
Physical condition	1. No chronic diseases 2. Chronic diseases	123 207	37.3 62.7
Psychological condition	1. No depression 2. Depression	285 45	86.4 13.6
Diet status	1. Meat-eater 2. Vegetarian	313 17	94.8 5.2
Exercise	1. No regular exercise 2. Regular exercise	185 145	56.1 43.9
Smoking habits	1. Nonsmoker 2. Smoker	294 36	89.1 10.9
Alcohol consumption	1. Nondrinker 2. Drinker	271 59	82.1 17.9
Social activities	1. No social activities 2. Social activities	54 276	16.4 83.6
Family support	1. No family support 2. Family support	5 325	1.5 98.5
Economic sources	1. Family-provided 2. Personal savings 3. Pension	185 97 48	56.1 29.4 14.5
Living status	1. Living alone 2. Living with family	32 298	9.7 90.3
Reading activities	1. Nonreader 2. Reader	217 113	65.8 34.2

### 4.2. Cues to action

Older adults’ cues to action included in dementia prevention and health promotion constituted television 233 (70.6%), newspapers 61 (18.5%), websites 65 (19.7%), health care workers 77 (23.3%), and other information 42 (12.7%) (Table 2).

**Table 2:** Cues to action

N = 330	Items	Frequency	Percentage
Cues to action	1. Television 2. Newspapers 3. Websites 4. Health care workers 5. Other information	233 61 65 77 42	70.6 18.5 19.7 23.3 12.7

### 4.3. AD8 distribution

This survey study discovered older adults’ AD8 distribution. The AD1 responses of the older adults regarding difficulty in judgment were as follows: 26 (7.9%) answered yes, 279 (84.5%) answered no, and 25 (7.6%) answered unknown (Table 3). In terms of decreased interest in activities and hobbies, the AD2 indicated that 53 (16.1%) older adults showed yes, 264 (80.0%) showed no, and 13 (3.9%) were unknown (Table 3). AD3 concerned repeating the same questions, stories, and statements; 103 (31.2%) older adults answered yes, 221 (67.0%) answered no, and 6 (1.8%) answered unknown (Table 3). AD4 referred to the difficulty in learning how to use tools, equipment, and devices: 46 (13.9%) older adults answered yes, 274 (83.0%) answered no, and 10 (3.0%) answered unknown (Table 3). AD5 concerned forgetting the correct month and year: 33 (10.0%) older adults answered yes, 291 (88.2%) answered no, and 6 (1.8%) answered unknown (Table 3). AD6 referred to difficulty dealing with complex financial matters; 27 (8.2%) older adults replied yes, 287 (87.0%) replied no, and 16 (4.8%) replied unknown (Table 3). AD7 referred to difficulty remembering dates; 26 (7.9%) older adults answered yes, 288 (87.3%) answered no, and 16 (4.8%) answered unknown (Table 3). AD8 concerned problems with continuous thinking and memory; 68 (20.6%) older adults answered yes, 246 (74.5%) answered no, and 16 (4.8%) answered unknown (Table 3).

**Table 3:** AD8 distribution

N = 330	Items	Frequency	Percentage
AD1 Difficulty in judgment	1. Yes 2. No 3. Unknown	26 279 25	7.9 84.5 7.6
AD2 Decreased interest in activities and hobbies	1. Yes 2. No 3. Unknown	53 264 13	16.1 80.0 3.9
AD3 Repeat the same questions, stories, and statements	1. Yes 2. No 3. Unknown	103 221 6	31.2 67.0 1.8
AD4 Difficulty in learning how to use tools, equipment, and devices	1. Yes 2. No 3. Unknown	46 274 10	13.9 83.0 3.0
AD5 Forget the correct month and year	1. Yes 2. No 3. Unknown	33 291 6	10.0 88.2 1.8
AD6 Difficulty dealing with complex financial matters	1. Yes 2. No 3. Unknown	27 287 16	8.2 87.0 4.8
AD7 Difficulty remembering dates	1. Yes 2. No 3. Unknown	26 288 16	7.9 87.3 4.8
AD8 Problems with continuous thinking and memory	1. Yes 2. No 3. Unknown	68 246 16	20.6 74.5 4.8

### 4.4. Mean scores on health beliefs, self-efficacy, and behavioral intention

The results of the study showed that older adults had the following mean scores on the health beliefs model of perceived susceptibility 13.45 ± SD 2.34, perceived severity 13.54 ± SD 2.69, perceived benefits 16.57 ± SD 2.84, perceived barriers 8.20 ± SD 3.69, self-efficacy 16.96 ± SD 3.52, and behavioral intention 19.56 ± SD 3.51 (Table 4). The 95% confidence interval of the lower and upper limit is indicated in Table 4, as follows: perceived susceptibility between 13.20 and 13.70, perceived severity between 13.25 and 13.83, perceived benefits between 16.27 and 16.88, perceived barriers between 7.80 and 8.60, self-efficacy between 16.58 and 17.34, and behavioral intention between 19.18 and 19.94.

**Table 4:** Mean scores on health beliefs, self-efficacy, and behavioral intention

N = 330	Items	Mean	SD	95% Confidence interval	
				Lower limit	Upper limit
Perceived susceptibility	4	13.45	2.34	13.20	13.70
Perceived severity	4	13.54	2.69	13.25	13.83
Perceived benefits	4	16.57	2.84	16.27	16.88
Perceived barriers	4	8.20	3.69	7.80	8.60
Self-efficacy	5	16.96	3.52	16.58	17.34
Behavioral intention	5	19.56	3.51	19.18	19.94

### 4.5. Spearman’s rho correlation

Spearman’s rho correlation showed that 330 older adults’ perceived susceptibility was positively correlated with perceived severity (*r* = 0.202; *P* < .01), and perceived benefits (*r* = 0.222; *P* < .01) (Table 5). Moreover, perceived severity was positively associated with perceived benefits (*r* = 0.235; *P* < .01) (Table 5). In addition, perceived benefits were positively correlated with perceived barriers (*r* = −0.355; *P* < .01), self-efficacy (*r* = 0.301; *P* < .01), and behavioral intention (*r *= 0.448; *P* < .01) (Table 5). Older adults’ perceived barriers were positively associated with self-efficacy (*r* = −0.197; *P* < .01) and behavioral intention (*r* = −0.393; *P* < .01) (Table 5). Furthermore, self-efficacy was positively correlated with behavioral intention (*r* = 0.606; *P* < .01) (Table 5).

**Table 5:** Spearman’s rho correlation

N = 330	Perceived susceptibility	Perceived severity	Perceived benefits	Perceived barriers	Self-efficacy	Behavioral intention
Perceived susceptibility	1					
Perceived severity	.202*	1				
Perceived benefits	.222*	.235*	1			
Perceived barriers	−.050	.049	−.355*	1		
Self-efficacy	−.054	.028	.301*	−.197*	1	
Behavioral intention	.090	.062	.448*	−.393*	.606*	1

* *P* < .01.

### 4.6. Simple regression analysis of the results

Older adults’ demographic background included gender, age, marriage, children, educational level, religion, physical condition, psychological condition, diet status, exercise, smoking habits, alcohol consumption, social activities, family support, economic sources, living status, and reading activities. The variable results with unstandardized coefficients, beta estimates, standard error, standardized coefficients, beta distribution, and *t*-values are shown in Table 6. In the simple regression analysis of the results, older adults’ demographic background, health belief model on perceived susceptibility, perceived severity, perceived benefits, perceived barriers, and self-efficacy explained 56.1% of the variance in behavioral intention (*R^2^* = 0.561; *F* = 17.822; *P* < .001) for dementia prevention and health promotion (Table 6).

**Table 6:** Simple regression analysis of the results

Variables	Unstandardized coefficients		Standardized coefficients	*t*-values
	Beta estimates	SE(Standard error)	Beta distribution	
Constant	1.888	4.230		.446
Gender	−.402	.338	−.057	−1.189
Age	−.055	.196	−.012	−.279
Marriage	.319	.302	.046	1.056
Children	.480	1.296	.015	.370
Educational level	−.131	.299	−.019	−.437
Religion	−.121	.368	−.013	−.328
Physical condition	.465	.296	.064	1.572
Psychological condition	−.074	.409	−.007	−.180
Diet status	.357	.615	.022	.581
Exercise	.607	.290	.086	2.092*
Smoking habits	−.480	.479	−.043	−1.002
Alcohol consumption	−.230	.389	−.025	−.593
Social activities	−.083	.385	−.009	−.215
Family support	.898	1.113	.031	.807
Economic sources	.292	.193	.061	1.515
Living status	.045	.482	.004	.093
Reading activities	.269	.304	.036	.885
Perceived susceptibility	.074	.063	.049	1.171
Perceived severity	−.024	.054	−.018	−.441
Perceived benefits	.326	.055	.264	5.916**
Perceived barriers	−.166	.040	−.174	−4.189**
Self−efficacy	.522	.044	.524	11.815**
	R^2^ = 0.561			F = 17.822**

* *P* < .05.

** *P* < .001.

## 5. Discussion

In Taiwan’s society, people are more and more concerned regarding dementia prevention and health promotion. Many diseases may cause the risks of dementia, such as head injury, hypertension, hyperglycemia, hyperlipidemia, depression, and other diseases. After this study, older adults were concerned about health beliefs to do dementia prevention and health promotion in daily life. The researcher will continue to establish a health beliefs model on the 4 concepts of physical, mental, spiritual, and social health promotion and dementia prevention for coping aging process.^[5]^

Some studies use health belief models and knowledge to explore or survey dementia prevention with many people.^[37,38]^ In the article, it is a health-promoting lifestyle prediction model among adults for dementia prevention and is based on the health belief model.^[3]^ Another article, it is used a health concept for primary dementia prevention about proof concept study on beliefs, attitudes, and appreciation.^[7]^ This study used the health beliefs model to survey older adults with AD8, cues to action, health beliefs (perceived susceptibility, perceived severity, perceived benefits, and perceived barriers), self-efficacy, and behavioral intention for dementia prevention and health promotion in daily life.

An analysis of the results clearly revealed that older adults need to be equipped with health education on dementia prevention and health promotion to delay the aging process. Specifically, older adults should be educated about AD8, cues to action, health beliefs, self-efficacy, and behavioral intention to achieve and sustain a high quality of life. In terms of delaying the aging process, researchers recommend that older adults use multiple strategies, such as cognitive activity,^[18]^ regular exercise,^[24]^ Mediterranean Diet,^[22]^ fruits and vegetables,^[21]^ prevention of the 3-high chronic diseases,^[20]^ emotional maintenance,^[12]^ social interaction,^[26]^ and AD8 screening^[27]^ to prevent dementia and promote health.

The AD8 distribution showed that, out of 330 older adults, 7.9% had a change in AD1, 16.1% had a change in AD2, 31.2% had a change in AD3, 13.9% had a change in AD4, 10.0% had a change in AD5, 8.2% had a change in AD6, 7.9% had a change in AD7, and 20.6% had a change in AD8. The AD8 screening is a simple test for a change in one score. If older adults have a score greater than or equal to 2, they should seek a physician’s diagnostic evaluation for dementia prevention and health promotion.^[31–33]^ In accordance with this, after the survey study, the research team recommended that older adults with a score of 2 or higher on the AD8 needed to see a physician for dementia assessment, diagnosis, and treatment.

Utilizing a telephonic meeting is an early screening approach, without the need for face-to-face interaction, and can be conducted in a short period of time with 12 questions to predict the risk of dementia among older adults in communities.^[39]^ Lifestyle improvement is a method for preventing dementia and promoting health. Modifying health risk behaviors aims to change unhealthy lifestyles with a positive motivation to achieve a high quality of life.^[40]^ Positive cues to action are associated with lifestyle changes on a 10-item risk-reduction scale for dementia prevention.^[40]^ Positive knowledge, health beliefs, and health attitudes are the most important factors for older adults to be educated on dementia prevention in communities.^[41]^ Overall, older adults should be educated on dementia prevention associated with knowledge, cues to action, changing lifestyles, health motivation, and self-efficacy to achieve optimal behavioral intention.^[42]^

The study showed that dementia prevention can be achieved with healthcare approaches to improve older adults’ knowledge, self-efficacy, and behavioral intention.^[43]^ Health-care workers are responsible for teaching older adults about dementia prevention and health promotion, promoting self-efficacy, and reaching behavioral intentions in community settings.^[43]^ The results showed that older adults’ cues to action in dementia prevention and health promotion constituted 5 strategies: television, healthcare workers, websites, newspapers, and other information. First, television was found to be the optimal modality for dementia prevention and health promotion among older adults. Second, healthcare workers can be effective in communicating health education to older adults. Third, websites are popular communication tools that can provide valuable information to older adults. Indeed, older adults’ cues to action comprise many pipelines that can disseminate useful information on dementia prevention and health promotion in Taiwan.

Older adults with healthy lifestyles have fewer risk factors for dementia. Social network applications on smartphones are an effective modality that can improve self-efficacy to change unhealthy life habits through coach-supported health.^[44,45]^ Improving self-efficacy and decreasing depressive symptoms can improve cognitive function in older adults for dementia prevention.^[46]^ Memory and visual-motor integration among older adults have been shown to increase self-efficacy in improving cognitive function and preventing dementia.^[47]^ Overall, dementia prevention is associated with self-efficacy in reducing dementia risk and promoting health behaviors.^[48–50]^ Therefore, self-efficacy in older adults can achieve health promotion, dementia prevention, and healthy lifestyles.^[48–50]^

According to the study results, older adults’ mean scores on the health belief model were perceived susceptibility, 13.45; perceived severity, 13.54; perceived benefits, 16.57; perceived barriers, 8.20; self-efficacy, 16.96; and behavioral intention, 19.56. The analysis of the results showed that older adults’ behavioral intention, self-efficacy, and perceived benefits obtained higher mean scores, perceived barriers obtained lower mean scores, and perceived susceptibility and perceived severity obtained middle-to-high mean scores. Therefore, the research team recommends promoting older adults’ health beliefs model on perceived susceptibility, perceived severity, perceived benefits, perceived barriers, and self-efficacy to improve behavioral intention for dementia prevention and health promotion.

This study found that self-efficacy accounted for 34.2% of the variance in behavioral intention in terms of dementia prevention and health belief model.^[51]^ In the study results of simple regression analysis, older adults’ demographic background, health belief model on perceived susceptibility, perceived severity, perceived benefits, perceived barriers, and self-efficacy explained 56.1% of the variance in behavioral intention for dementia prevention and health promotion. From this point result, 56.1% of behavioral intention indicated that predicted older adults’ healthy behaviors in daily life.

In summary, the study purpose was to survey older adults’ demographic background, AD8, cues to action, health beliefs, self-efficacy, and behavioral intention. The results of the study showed satisfactory results and expectations to meet the research purpose. In accordance with these results, the research team will continue to promote older adults’ health beliefs, self-efficacy, and behavioral intention to achieve dementia prevention and health promotion. The authors of the study are located in Kaohsiung, Pingtung, and Taipei city. We use LINE, Email, and Gmail, to meet and contact each other. In the future study, the research group will develop the strengths of the present study with many strategies, such as beverages, music, pictures, and scriptures in regarding interventions on physical, mental, spiritual, and social health promotion among older adults for keeping dementia prevention.^[5]^

## 6. Limitations

The main limitation of this study is that the participants were limited to 330 older adults above 55 years old in Kaohsiung-Pingtung City, Taiwan, ROC. To increase the generalizability and robustness of the results, similar studies could be conducted with diverse populations and other geographical settings.

## 7. Conclusions

The conclusions of the study indicated that older adults’ demographic background, AD8, cues to action, health beliefs, self-efficacy, and behavioral intention constituted the main factors for effective dementia prevention and health promotion. A total of 330 convenience samples among older adults participated in a cross-sectional survey study. The study findings were as follows: older adults’ cues to action included in dementia prevention and health promotion constituted television 70.6%, newspapers 18.5%, websites 19.7%, health care workers 23.3%, and other information 12.7%; older adults with a score of 2 or higher on the AD8 were recommended to see a neurologist for early dementia assessment, diagnosis, and treatment; older adults’ mean scores on the health beliefs model were perceived susceptibility 13.45, perceived severity 13.54, perceived benefits 16.57, perceived barriers 8.20, self-efficacy 16.96, and behavioral intention, 19.56; older adults’ demographic backgrounds, health beliefs on the perceived susceptibility, perceived severity, perceived benefits, perceived barriers, and self-efficacy explained 56.1% of the variance in behavioral intention. In the future, the research team will continue to explore older adults’ dementia prevention and develop many strategies on health promotion, as well as slowing the aging brain process.

## Author contributions

**Conceptualization:** Fu-Ju Tsai, Sheng-Wei Shen.

**Data curation:** Fu-Ju Tsai.

**Formal analysis:** Fu-Ju Tsai.

**Funding acquisition:** Fu-Ju Tsai.

**Investigation:** Fu-Ju Tsai.

**Methodology:** Fu-Ju Tsai, Sheng-Wei Shen.

**Project administration:** Fu-Ju Tsai.

**Resources:** Fu-Ju Tsai.

**Software:** Fu-Ju Tsai.

**Writing – original draft:** Fu-Ju Tsai.

**Writing – review & editing:** Fu-Ju Tsai, Sheng-Wei Shen.

**Supervision:** Sheng-Wei Shen, Yih-Jin Hu, Chie-Chien Tseng.

**Validation:** Sheng-Wei Shen, Yih-Jin Hu, Chie-Chien Tseng.

**Visualization:** Sheng-Wei Shen, Yih-Jin Hu, Chie-Chien Tseng.
